# Evaluation of the efficacy and safety of the augmented rectangle technique for Billroth-I reconstruction in laparoscopic distal gastrectomy for gastric cancer: comparison with Roux-en-Y reconstruction

**DOI:** 10.1007/s00464-025-12281-4

**Published:** 2025-10-06

**Authors:** Shinya Mikami, Takeharu Enomoto, Jin Shimada, Yasuhito Hisatsune, Yoshitsugu Tsukamoto, Masaki Hiwatari, Sae Kimura, Tsunehisa Matsushita, Osamu Saji, Takehito Otsubo

**Affiliations:** Department of Gastroenterological and General Surgery, St. Marianna University School of Medicine, 2-16-1 Sugao Miyamae-Ku, Kawasaki-Shi, Kanagawa 216-8511 Japan

**Keywords:** Laparoscopic distal gastrectomy, Billroth-I reconstruction, Augmented rectangle technique, Roux-en-Y reconstruction, Anastomosis, Postoperative outcomes

## Abstract

**Background:**

Laparoscopic distal gastrectomy confers a large rectangular anastomotic stoma, which can be repaired with an augmented rectangle technique anastomosis in an intracorporeal Billroth-I reconstruction. We aimed to evaluate the safety and feasibility of the augmented rectangle technique (ART) for anastomosis.

**Methods:**

We retrospectively compared the surgical outcomes and postoperative nutritional statuses of patients who received Billroth-I (*n* = 285; ART group) or Roux-en-Y (*n* = 72; R-Y group) reconstruction between January 2013 and December 2023 following laparoscopic distal gastrectomy for gastric cancer at our hospital. Furthermore, postoperative changes in nutritional status of patients who did or did not receive postoperative chemotherapy were compared for at least 1 year in both ART and R-Y groups (*n* = 198 and *n* = 42, respectively). Results: Among the 357 participants, patients in the ART (196 men and 89 women) and R-Y (54 men and 18 women) groups had a median age of 71 and 74 years, respectively. The ART group, compared to the R-Y group, had significantly shorter operative time (260 min vs. 326 min), less blood loss (32 mL vs. 98 mL), and shorter postoperative hospital stay (9 days vs. 10 days) (all *p* < 0.001). There was no significant intergroup difference in anastomosis-related complications or anastomotic leakage [ART group vs. R-Y group: 3 (1.1%) vs. 1 (1.4%); *p* = 0.814 for both]. No patient had anastomotic stenosis or hemorrhage. At 3, 6, and 12 months, there was no significant intergroup difference in the hemoglobin and serum albumin levels or the postoperative weight change (ART group vs R-Y group: − 6.7% vs. − 7.9%, *p* = 0.262; − 7.1% vs. − 8.3%, *p* = 0.726; and − 7.0% vs. − 7.9%, *p* = 0.905, respectively), despite a favorable tendency in the ART group.

**Conclusions:**

The augmented rectangle technique of anastomosis is a safe, effective technique for total intracorporeal Billroth-I reconstruction and confers favorable postoperative outcomes and satisfactory oral intake.

**Supplementary Information:**

The online version contains supplementary material available at 10.1007/s00464-025-12281-4.

Gastric cancer (GC) was the fifth most common cancer and the fourth leading cause of cancer-related deaths worldwide in 2020 [1]. As surgical resection is the primary treatment modality for GC, surgical techniques that reduce postoperative burden and optimize the patient’s quality of life are warranted. Laparoscopic-assisted distal gastrectomy (LADG) for GC was first performed in Japan in 1994 [2]. Since then, laparoscopic surgery has been widely employed for patients with GC and has resulted in satisfactory surgical outcomes. Large-scale randomized controlled trials (RCTs) in East Asian countries have evaluated the safety and effectiveness of LADG or laparoscopic distal gastrectomy (LDG) for Stage Ⅰ GC [3, 4] whereas subsequent RCTs have investigated these aspects in advanced GC [5, 6]. In 2023, an RCT (JLSSG0901) in Japan demonstrated the non-inferiority of LADG to open distal gastrectomy. Therefore, LADG was recommended as a surgical treatment option in cases with cStage II/III advanced GC that require distal gastrectomy with D2 lymph node dissection [7].

Distal gastrectomy is the most commonly performed procedure for GC located in the middle or lower stomach. However, the selection of the optimal reconstructive procedure following a distal gastrectomy has not been standardized and remains debatable in the absence of consensus. The methods for post-LDG intracorporeal anastomosis include delta-shaped anastomosis (DA), Billroth-I (B-I), and Roux-en-Y (R-Y) [8–12]. Kanaya et al. [8] reported that DA comprises a B-I anastomotic technique that involves the utilization of two linear staplers in LDG and is the standard procedure for post-LDG intracorporeal B-I reconstruction in many countries, including Asia [8–10]. Despite its proven safety and feasibility, DA is associated with postoperative complications, such as anastomotic leakage or anastomotic stenosis. Subsequently, improvements to DA that have enhanced the usefulness of intracorporeal B-I reconstruction methods have been reported, including the augmented rectangle technique (ART) [13], intracorporeal triangle anastomotic technique (INTACT) [14, 15], and the Modified Delta-shaped anastomosis (Mod-DA) [16–18]. Fukunaga et al., in 2013, invented the ART anastomosis for intracorporeal B-I reconstruction with a three-linear stapler method, and this technique is characterized by a large rectangular anastomotic stoma; moreover, the duodenal stump is detached during anastomosis and results in an end-to-end anastomosis. In 2018, a study demonstrated the feasibility and safety of the ART anastomosis, which had a low incidence of anastomosis-related complications [13]. The main feature of ART is that the duodenal stump is resected and that a T-shaped stapler intersection region is not created at the anastomosis. To our knowledge, this is the first study to compare the differences in the postoperative outcomes of the post-LDG ART and R-Y.

This study aimed to compare the surgical outcomes and postoperative nutritional status after B-I using ART anastomosis with those of R-Y following LDG and to evaluate the feasibility of ART anastomosis.

## Materials and methods

### Participants

Between January 2013 and December 2023, 370 patients underwent LDG for GC at St. Marianna University Hospital and, among them, 290 patients underwent B-I reconstruction with ART. After excluding five patients who underwent simultaneous colorectal cancer surgery, we retrospectively examined the clinical characteristics and surgical outcomes of 285 patients in the ART group. These data were accessed for research purposes from January 1, 2024, to December 31, 2024. The authors had no access to any information that could identify individual participants during or after data collection. Furthermore, among the 80 patients who underwent R-Y reconstruction during the same period, we excluded eight patients who underwent subtotal gastrectomy for tumors in the U-region and had an extremely small residual stomach volume, and enrolled 72 patients in the R-Y group. Moreover, we compared the differences in postoperative nutritional status between the ART and R-Y groups in patients who did not receive postoperative chemotherapy and had undergone surgery more than 1 year before the study (*n* = 198 and *n* = 42, respectively).

**Fig. 1:**
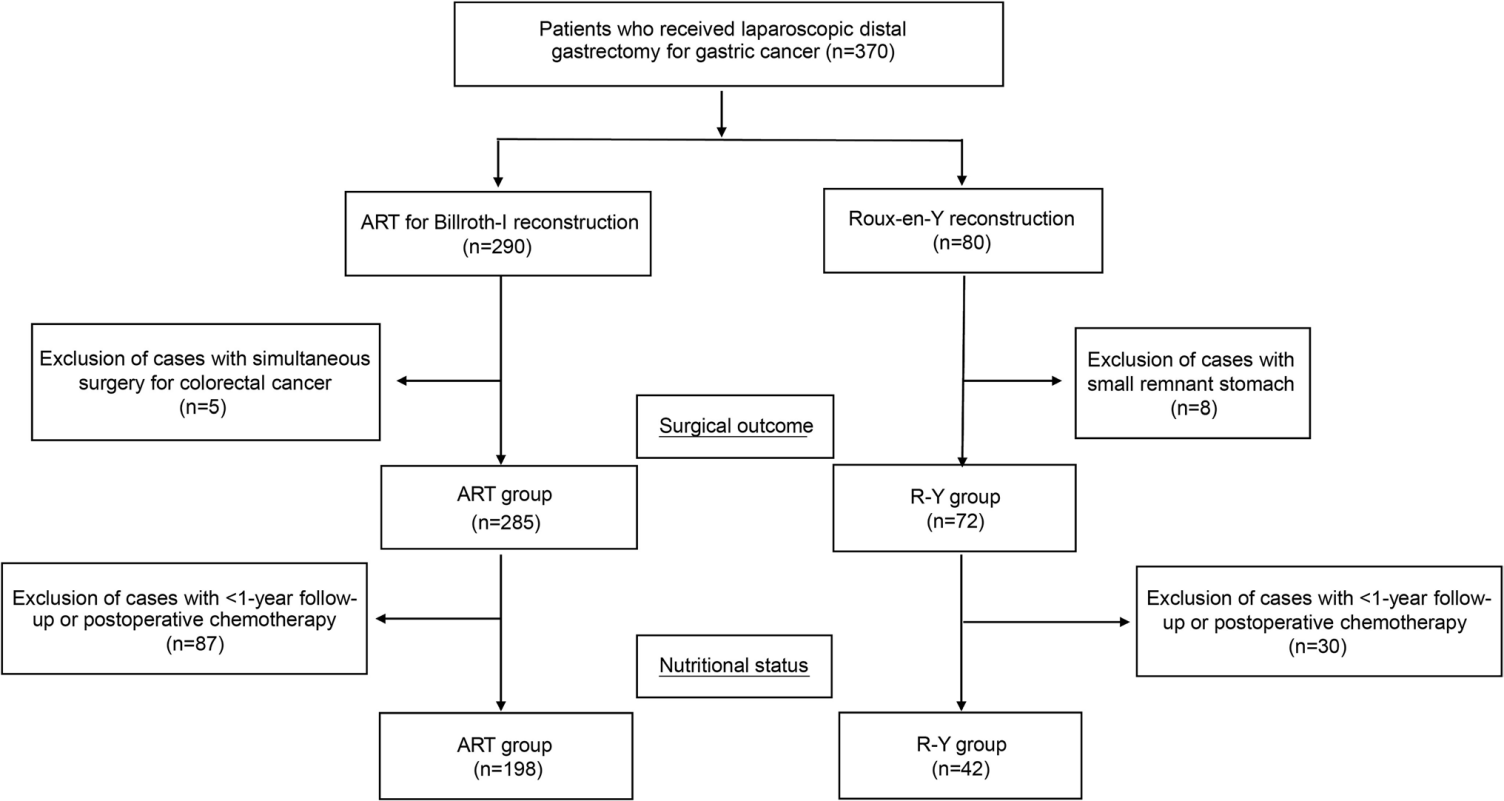
Flowchart of patient selection. Among 370 patients who underwent LDG between January 2013 and December 2023, we identified patients who underwent either ART for B-I reconstruction or R-Y and examined surgical outcomes in 285 and 72 patients and nutritional status in 198 and 42 patients, respectively. *ART* augmented rectangle technique; *LDG* laparoscopic distal gastrectomy; *R-Y* Roux-en-Y

Preoperatively and at 3, 6, and 12 months postoperatively, nutritional parameters as well as body weight, body mass index (BMI), and hemoglobin and albumin levels were assessed. Figure 1 presents a flowchart of the patient selection and disposition.

This study was approved by the Institutional Review Board of the St. Marianna University School of Medicine Bioethics Committee (approval number: 6110). The need for informed consent was waived because of the retrospective nature of the study as well as the analysis of the anonymized data. All procedures were performed in accordance with the relevant guidelines and regulations. Disease staging was undertaken in accordance with the TNM Classification (8th Edition) [19] whereas postoperative complications were graded according to the Clavien–Dindo classification system [20].

### Surgical procedure

#### Selection of reconstruction methods in distal gastrectomy

In our hospital, distal gastrectomy is performed with B-I reconstruction, whereas the R-Y reconstruction is performed only in cases with a small residual stomach volume, strained anastomosis, tumor located near or invading the duodenum, or a more than moderate hiatal esophageal hernia.

### ART for Billroth-I reconstruction

By using a 60-mm endoscopic linear stapler (ELS), the duodenum was transected near the pyloric ring from the greater curvature to the lesser curvature. After lymph node dissection, confirming the site of the lesion, and marking the area to be dissected, we used ELS to dissect the oral margin of the stomach from the greater to the lesser curvature; two-thirds of the stomach was resected and removed through a small incision in the umbilical port. A small hole was created in the greater curvature of the duodenal and gastric transection lines, the cartridge side of the 60-mm ELS was inserted parallel to the suture line on the posterior wall of the stomach, and the forked side of the ELS was guided to the posterior wall of the duodenum. Then, the device was closed and fired.

**Fig. 2:**
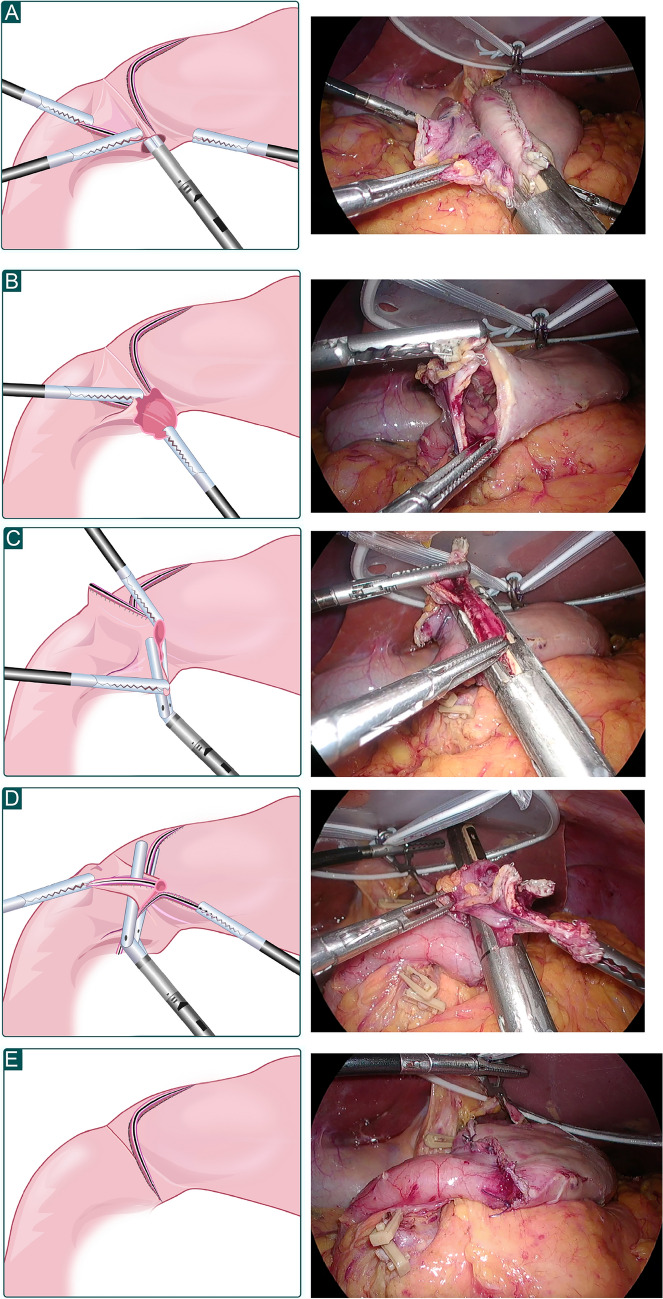
Schematic outline of the ART anastomosis methods. **A** Both posterior walls of the remnant stomach and the duodenum are anastomosed using a 60-mm ELS. **B** A V-shaped anastomosis is created on the posterior wall of the gastroduodenal anastomosis. **C** The common entry hole in the anterior wall of the greater curvature is sutured using a 30-mm ELS from the greater curvature to immediately before the duodenal stump. **D** The anterior wall of the lesser curvature is sutured using a 60-mm ELS with the tip overlapping the suture line of the gastrectomy, including the duodenal stump. **E** This created a square end-to-end anastomosis with the remnant of the gastroduodenal anastomosis. *ART* augmented rectangle technique; *ELS* endoscopic linear stapler

A V-shaped anastomosis was created at the posterior wall of the gastroduodenal site. The anterior wall was externally sutured using two sutures: one each on the side of the greater and lesser curvatures. First, the common entry hole in the anterior wall of the greater curvature was sutured using a 30-mm ELS from the greater curvature to immediately before the duodenal stump. Next, the anterior wall of the lesser curvature was sutured using a 60-mm ELS with the tip overlapping the suture line of the gastrectomy, including the duodenal stump. This created a square end-to-end anastomosis with the remnant of the gastroduodenal anastomosis (Fig. 2).

### Roux-en-Y reconstruction

After lymph node dissection, two-thirds of the stomach was resected and removed through a small incision in the umbilical port. The jejunum, which was situated approximately 25 cm distal to the ligament of Treitz, was marked laparoscopically, moved extracorporeally, and transected using a linear stapler. Next, 30 cm distal to the gastrojejunostomy, a jejunojejunostomy was performed wherein, using a 60-mm ELS, the oral portion of the jejunum was anastomosed side to side, and the entry hole was closed using sutures. To prevent an internal hernia, the jejunal mesentery was sutured and closed with a non-absorbable suture. In an antecolic manner, the distal jejunal stump was pulled up to the remnant stomach, and using a 60-mm ELS, a gastrojejunostomy was performed by creating a side-to-side isoperistaltic anastomosis between the distal jejunal stump and the greater curvature of the remnant stomach. The entry hole was closed using continuous barbed sutures. Finally, the Petersen’s defect was closed with continuous sutures using a resorbable suture.

### Postoperative management

At our hospital, all patients were managed according to the Enhanced Recovery After Surgery (ERAS) concept-based critical pathway, and postoperative management was performed in the same manner. During surgery, a drain was inserted posterior to the anastomotic site for all patients. The nasogastric tube was removed immediately after surgery. Ambulation training was initiated on postoperative day (POD)1, and water intake was allowed, starting on POD1. The patients were started on an oral nutritional supplement on POD3 and a soft diet on POD4. The drain was removed on POD5, followed by patient discharge between PODs 7 and 9. The quality of life was assessed using a specific questionnaire at ≥ 6 months postoperatively. Thereafter, the patients were regularly followed up on an outpatient basis.

Dumping syndrome was evaluated using the Sigstad’s score [21]. A Sigstad’s score of ≥ 7 indicated dumping syndrome, 4–7 indicated borderline, and ≤ 4 indicated non-dumping syndrome.

### Statistical analysis

All data were analyzed using JMP Pro 16 software (SAS Institute Inc., Cary, NC, USA). Statistical analyses of intergroup differences were performed using the Mann–Whitney U and Fisher’s exact tests. For all analyses, *p* < 0.05 was considered statistically significant.

## Results

### Participant’s characteristics

Table 1 presents the clinicopathological characteristics of the participants, including sex, age, BMI, comorbidities, tumor location, histological type, presence of ESD, and the clinical stage (TNM classification). The ART and R-Y groups included 285 and 72 patients, respectively.

**Table 1:** Clinicopathological characteristics of the participants

	ART group (*n* = 285)	R-Y group (*n* = 72)	*p*-value
Sex			0.303
Male	196 (68.8%)	54 (75%)	
Female	89 (31.2%)	18 (25%)	
Age (years) ^a^	71 (31–93)	74 (37–91)	0.044
BMI (kg/m^2^) ^a^	22.2 (13.5–35.4)	22.9 (16.9–32.3)	0.022
ASA-PS			0.423
Class I	29 (10.2%)	4 (5.6%)	
Class II	238 (83.5%)	62 (86.1%)	
Class III	18 (6.3%)	6 (8.3%)	
Comorbidity			0.378
Presence	179 (62.8%)	49 (68.1%)	
Absence	106 (37.2%)	23 (31.9%)	
Tumor location			0.446
Upper	0 (0%)	0 (0%)	
Middle	138 (48.4%)	31 (43.1%)	
Lower	147 (51.6%)	41 (56.9%)	
Histological type			0.304
Differentiated	159 (55.8%)	45 (62.5%)	
Undifferentiated	126 (44.2%)	27 (37.5%)	
ESD			0.006
Presence	35 (12.3%)	1 (1.4%)	
Absence	250 (87.7%)	71 (98.6%)	
Clinical T			0.006
T1	143 (50.2%)	23 (31.9%)	
T2–T4	142 (49.8%)	49 (68.1%)	
Clinical N			0.036
N0	240 (84.2%)	53 (73.6%)	
N1–N3	45 (15.8%)	19 (26.4%)	
Clinical stage			
I	196 (68.8%)	34 (47.3%)	0.001
IIA	16 (5.6%)	5 (6.9%)	
IIB	48 (16.8%)	16 (22.2%)	
III	25 (8.8%)	17 (23.6%)	

*ART* augmented rectangle technique; *BMI* Body mass index; *ASA-PS* American Society of Anesthesiologists Physical Status Classification; *ESD* Endoscopic submucosal resection; *R-Y* Roux-en-Y

^a^Data are expressed as median (range)

### Surgical outcomes

**Fig. 3:**
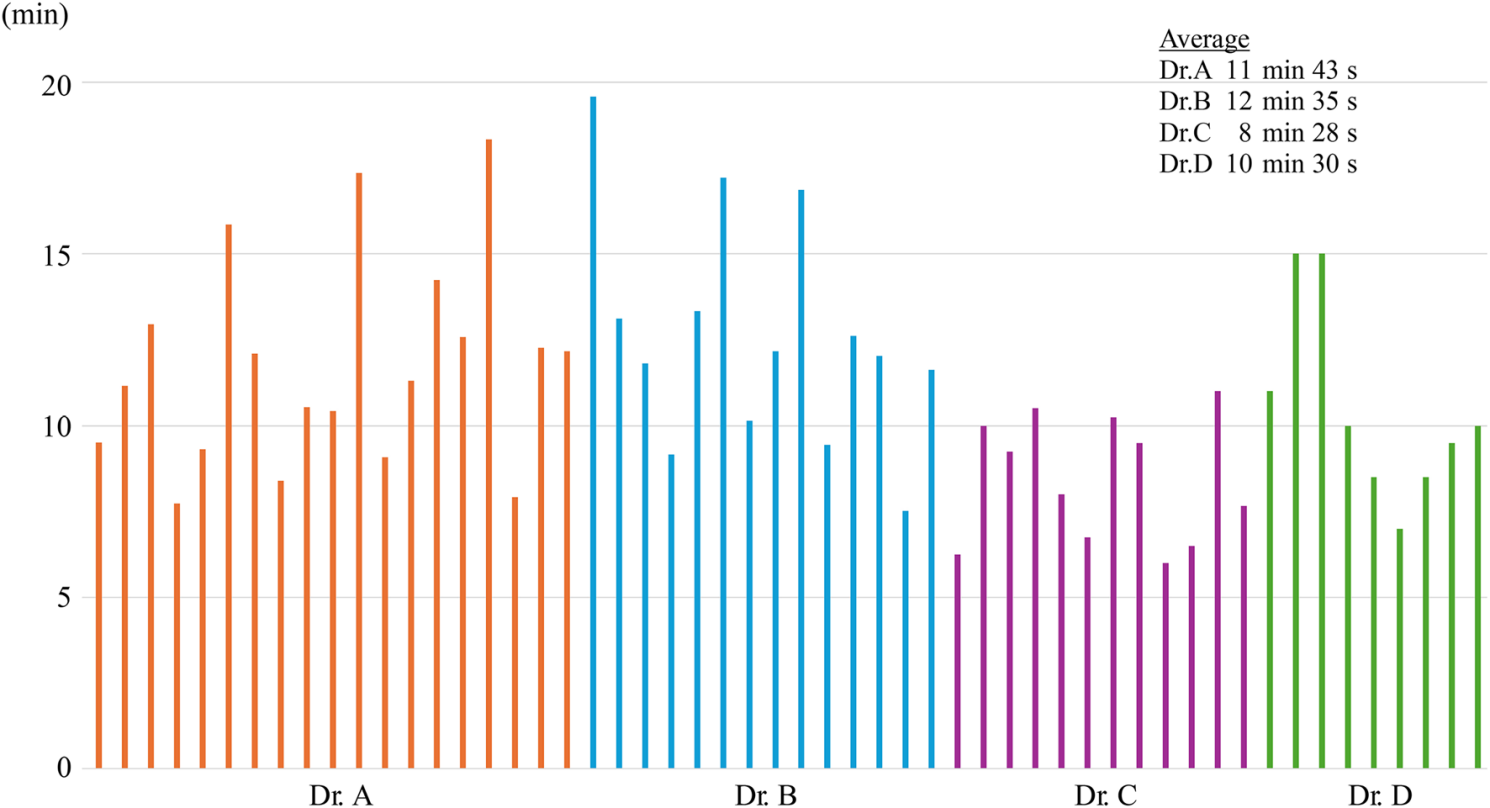
Anastomosis time of ART. The anastomosis times for four trainees over the past 5 years are indicated. *ART* augmented rectangle technique

Data on surgical outcomes are shown in Table 2. Compared to the R-Y group, the ART group had a significantly shorter operative time (260 min vs. 326 min), less blood loss (32 mL vs. 98 mL), and a shorter postoperative hospital stay (9 days vs. 10 days; all *p* < 0.001). No conversion to open surgery was performed. The anastomosis times of ART for four trainees over the past 5 years are shown in Fig. 3. The average anastomosis time for the four trainees was 11 min 2 s (range: 6 min 2 s–19 min 35 s).

**Table 2:** Surgical outcomes

	ART group (n = 285)	R-Y group (n = 72)	*p-*value
Lymph node dissection			0.058
D1	3 (1.1%)	0 (0%)	
D1 +	143 (50.2%)	26 (36.1%)	
D2	139 (48.7%)	46 (63.9%)	
Operation time (minutes) ^a^	260 (105–605)	326 (175–564)	< 0.001
Intraoperative blood loss (mL) ^a^	32 (3–1332)	98 (5–1372)	< 0.001
Conversion to open surgery	0 (0%)	0 (0%)	
R0 resection	285 (100%)	72 (100%)	N/A
Postoperative complications			
Anastomosis-related complications	3 (1.1%)	1 (1.4%)	0.814
Anastomotic leakage (CD grade ≥ II)	3 (1.1%)	1 (1.4%)	0.814
Anastomotic stenosis (any grade)	0 (0%)	0 (0%)	N/A
Anastomotic hemorrhage (any grade)	0 (0%)	0 (0%)	N/A
Delayed gastric emptying (any grade)	0 (0%)	1 (1.4%)	0.202
Pancreatic fistula (CD grade ≥ IIIa)	5 (1.8%)	2 (2.8%)	0.576
Intra-abdominal infection (CD grade ≥ II)	5 (1.8%)	2 (2.8%)	0.576
Intra-abdominal hemorrhage (CD grade ≥ IIIa)	2 (0.7%)	0 (0%)	1.000
Wound infection (CD grade ≥ II)	1(0.4%)	1 (1.4%)	0.363
Others (CD grade ≥ II)	6 (2.1%)	5 (7.0%)	0.034
Postoperative hospital stay (days) ^a^	9 (6–78)	10 (7–79)	< 0.001
Mortality	0 (0%)	0 (0%)	N/A
Postoperative chemotherapy			0.095
Presence	43 (15.1%)	17 (23.6%)	
Absence	242 (84.9%)	55 (76.4%)	
Pathological stage			0.014
IA	153 (53.6%)	28 (38.9%)	
IB	45 (15.8%)	10 (13.9%)	
IIA	25 (8.8%)	13 (18.0%)	
IIB	23 (8.1%)	11 (15.3%)	
IIIA	27 (9.5%)	3 (4.2%)	
IIIB	9 (3.1%)	6 (8.3%)	
IIIC	3 (1.1%)	1 (1.4%)	

*ART* augmented rectangle technique; *CD* Clavien–Dindo classification; *N/A* Not applicable; *R-Y* Roux-en-Y

^a^Data are expressed as median (range)

There was no significant intergroup difference in anastomosis-related complications or anastomotic leakage [ART group vs. R-Y group: 3 (1.1%) vs. 1 (1.4%); *p* = 0.814 for both]. No patient had anastomotic stenosis or hemorrhage. In all cases of anastomotic leakage, a pancreatic fistula posed a complication; however, there was no significant intergroup difference in pancreatic fistula [ART group vs. R-Y group: 5 (1.8%) vs. 2 (2.8%); *p* = 0.576].

No significant intergroup difference in delayed gastric emptying was observed [ART group vs. R-Y group: 0 (0%) vs. 1 (1.4%); *p* = 0.202]. Other events (CD Grade ≥ Ⅱ) occurred more frequently in the R-Y group than in the ART group (7% vs. 2.1%, *p* = 0.034). Moreover, the pathological stage was more advanced in the R-Y group than in the ART group (*p* = 0.014). Table 3 shows the details of complications categorized according to the Clavien–Dindo classification.

**Table 3:** Changes in the postoperative nutritional status

Complication		Grade 1	Grade 2	Grade 3a	Grade 3b	Grade 4a	Grade 4b	Grade 5
**ART**								
Anastomotic leakage, *n*				3				
Pancreatic fistula, *n*			1	3	1			
Intra-abdominal infection, *n*			3	2				
Intra-abdominal hemorrhage, *n*				1	1			
Wound infection, *n*		2	1					
Ileus*, n*		1						
Chylous ascites*, n*		1						
Duodenal obstruction*, n*				1				
Pneumonia, *n*			1					
Cerebral infarction*, n*				1				
Cholecystitis, *n*			1					
Prostatitis, *n*			1					
Urinary retention,* n*			1					
**R-Y**								
Anastomotic leakage, *n*					1			
Pancreatic fistula, *n*			1	2				
Intra-abdominal infection, *n*			2					
Wound infection, *n*			1					
Delayed gastric emptying, n			1					
Chylous ascites			1					
Pneumonia, *n*			2					
Urinary tract infection, *n*			1					
Heart failure, *n*						1		

### Nutritional status

**Fig. 4:**
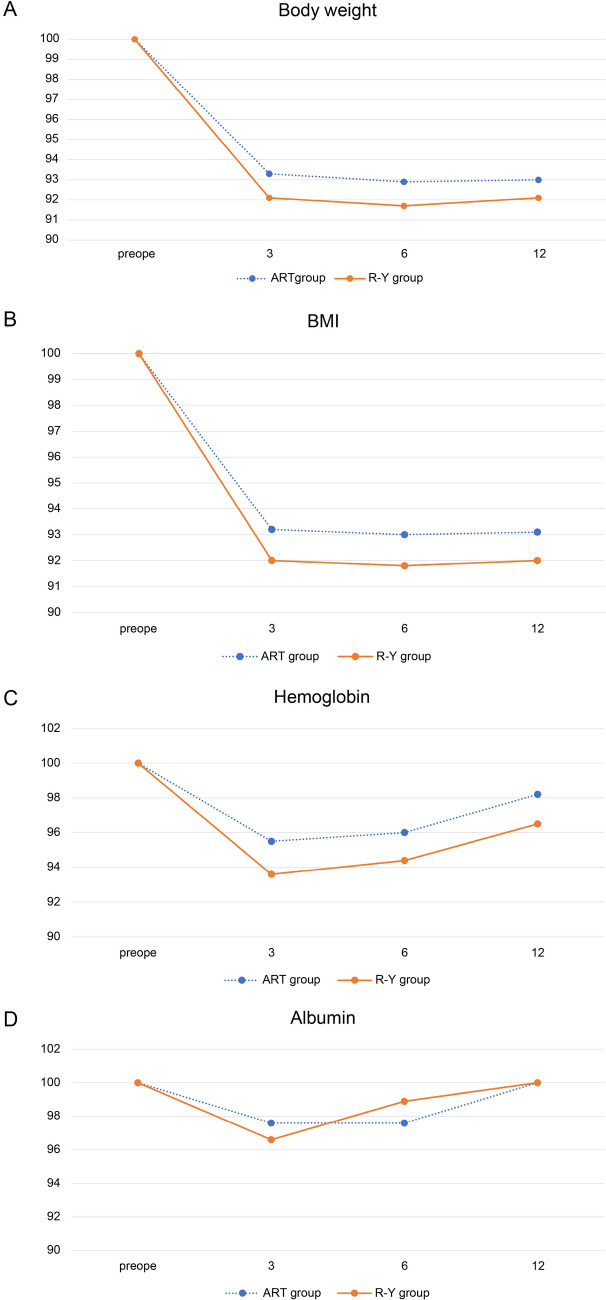
Changes in the postoperative nutritional status. **A** Change in body weight (kg); **B** Change in body mass index (kg/m^2^); **C** Change in serum hemoglobin (g/dL); **D** Change in serum albumin (g/dL). *Horizontal axis*: time from the operation. *Vertical axis*: median % at 3, 6, and 12 months, with a preoperative value of 100%. Preope: preoperative

Table 4 and Fig. 4 depict the intergroup differences in the changes in nutritional parameters at 3, 6, and 12 months postoperatively.

**Table 4:** Changes in the postoperative nutritional status

	ART group	R-Y group	*p*-value
A. Body weight			
POM 3	93.3% (76.6–117.6%)	92.1% (71.9–115.7%)	0.262
POM 6	92.9% (82.2–114.4%)	91.7% (73.1–114.3%)	0.726
POM 12	93.0% (82.4–117.6%)	92.1% (73.6–118.6%)	0.905
B. BMI			
POM 3	93.2% (76.4–117.8%)	92.0% (71.7–115.3%)	0.251
POM 6	93.0% (75.0–114.1%)	91.8% (73.0–114.0%)	0.710
POM 12	93.1% (71.7–117.8%)	92.0% (73.5–118.3%)	0.923
C. Hemoglobin			
POM 3	95.5% (68.1–146.7%)	93.6% (71.7–133.7%)	0.577
POM 6	96.0% (61.1–145.6%)	94.4% (78.7–145.4%)	0.529
POM 12	98.2% (79.5–150%)	96.5% (73.6–146.4%)	0.310
D. Albumin			
POM 3	97.6% (72.9–134.4%)	96.6% (67.4–11.8%)	0.263
POM 6	97.6% (82.2–137.5%)	98.9% (86.0–113.5%)	0.819
POM 12	100% (78.4–131.3%)	100% (79.1–113.5%)	0.877

**A** Change in body weight (kg); **B** Change in body mass index (kg/m^2^); **C** Change in serum hemoglobin (g/dL); **D** Change in serum albumin (g/dL). *ART* augmented rectangle technique; *POM* Postoperative month; *R-Y* Roux-en-Y

At 3, 6, and 12 months, the postoperative weight change showed a favorable, but nonsignificant, tendency in the ART group (ART group vs. R-Y group: − 6.7% vs. − 7.9%, *p* = 0.262; − 7.1% vs. − 8.3%, *p* = 0.726; and − 7.0% vs. − 7.9%, *p* = 0.905, respectively; Table 4 and Fig. 4A). Postoperative changes in BMI at 3, 6, and 12 months were similar to those of body weight (ART group vs. R-Y group: − 6.8% vs. − 8%, *p* = 0.251; − 7% vs. − 8.2%, *p* = 0.710; and − 6.9% vs. − 8%, *p* = 0.923, respectively; Table 4B and Fig. 4B).

**Fig. 5:**
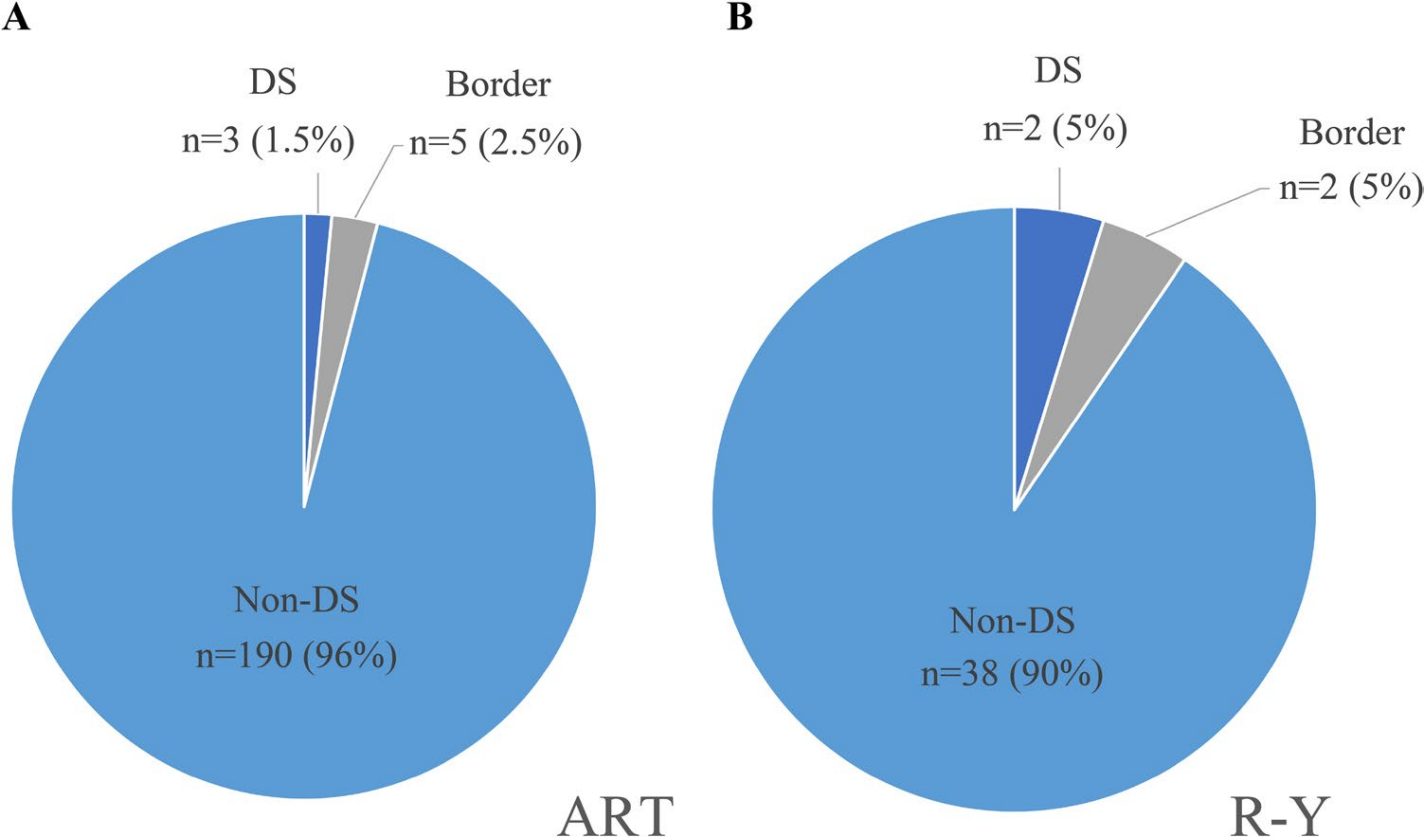
The incidence of dumping syndrome in the ART and R-Y groups. Dumping syndrome was evaluated using Sigstad’s score. **A** ART group at 1 year after surgery; **B** R-Y group at 1 year after surgery. DS dumping syndrome; Border borderline dumping syndrome

In both groups, body weight and BMI were lowest at 6 months postoperatively. At 3, 6, and 12 months, the postoperative hemoglobin levels in the ART group were lower than those in the R-Y group (− 4.5% vs. − 6.4%, *p* = 0.577; − 4.0% vs. − 5.6%, *p* = 0.529; and − 1.8% vs. − 3.5%, *p* = 0.310); however, the intergroup difference was not statistically significant (Table 4C and Fig. 4C). There was no significant intergroup difference in the serum albumin levels at 3, 6, and 12 months (Table 4D and Fig. 4D). Dumping syndrome occurred in 3 (1.5%) and 2 patients (4.7%) in the ART and R-Y groups, respectively, at 1 year after surgery (*p* = 0.18) (Fig. 5).

## Discussion

Since it was first performed in Japan in 1994 [2], LADG for GC has been widely employed in East Asian countries with a high incidence of GC, owing to the preference for minimally invasive surgery [3, 4]. The methods for post-LDG intracorporeal anastomosis include DA and R-Y [8–12]. In 2002, Kanaya et al. [8] reported a DA method that involved the use of two linear staplers for totally intracorporeal B-I reconstruction in LDG. In East Asia and other countries, DA is the most commonly performed anastomosis and, because of its relative simplicity, is the standard procedure for post-LDG intracorporeal B-I reconstruction [8, 9]. However, there are two major concerns regarding DA: first, two staple lines cross each other and, second, the fact that staples remain in the corner of the duodenal stump underscores the possibility of anastomosis-related complications, such as anastomotic leakage and stenosis [13–18]. The incidences of post-DA anastomotic leakage and anastomotic stenosis are 0.42–8.5% [16] and 0.8–2.4% [22], respectively. Therefore, several reconstructive methods, such as ART [13], INTACT [14, 15], and Mod-DA [16–18], have been developed as totally intracorporeal B-I reconstructive methods that overcome the limitations of DA.

The differences between ART and DA surgical techniques are as follows. First, during duodenal dissection, DA is performed from the posterior wall to the anterior wall, whereas ART is performed from the greater curvature to the lesser curvature. Second, in DA, the distance between the margin of the gastric remnant and the anastomotic site of the posterior wall of the remnant stomach prevents the development of an ischemic area in the remnant stomach. However, ART constitutes an anastomosis that ensures close contact between the margin of the gastric remnant and the posterior gastric wall wherein the last stapler is placed after removing the duodenal stump and ischemic gastric tissue remnant. Third, DA uses a single stapler to close the common entry hole without a duodenal stump and ensures traction with a stay suture. However, ART uses two staplers to close the common entry hole that contains the duodenal stump while applying forceps traction without a stay suture. Fourth, there are two T-shaped stapler intersections in DA, whereas ART uses two staplers for the closure of the common entry hole while including the duodenal stump. Theoretically, compared to the triangular anastomotic stoma in DA, the large quadrangular anastomotic stomas in ART are wider. Therefore, in terms of anastomotic stenosis, distortion, and gastric emptying, ART has significant advantages over other B-I reconstructive methods. Furthermore, the resection of the duodenal stump creates a true end-to-end anastomosis, and overlapping the staplers prevents the formation of a T-shaped stapler intersection, which is a major characteristic of this procedure [13, 22, 23]. The abovementioned features of ART prevent anastomotic leakages due to ischemic areas, which are a concern in DA. Furthermore, by preventing weak duodenal peristalsis owing to insufficient blood flow at the duodenal stump, ART ensures superior food passage through the anastomosis than that with DA [15, 18]. Fukunaga et al. reported that none of their patients who underwent ART had delayed gastric emptying, anastomotic stenosis, or anastomotic distortion. Thus, ART simplifies the technique, shortens the anastomosis time, and reduces the risk of leakage of intestinal contents into the abdominal cavity as no stay sutures are used for closure of the common entry hole. In this study, notably, the average anastomosis time for the four recent trainees was 11 min 2 s. The time plateaued at around 10 min after gaining extra experience. Therefore, it is considered a relatively simple technique that can be mastered in a short period. In this study, the incidence of anastomotic leakage and anastomosis-related complications was 1.1% in the ART group, and no patient had anastomotic stenosis or experienced delayed gastric emptying. Compression of the pancreas during suprapancreatic lymph node dissection is a major cause of pancreatic fistula [24, 25]. Therefore, since 2018, our hospital has improved our procedure to avoid direct compression of the pancreas with forceps. Instead, gentle compression is applied using a gauze and has consequently reduced the incidence of pancreatic fistula.

In this study, a pancreatic fistula occurred in all three patients (1.1%) with anastomotic leakage, and these patients experienced delayed suture failure during treatment of the pancreatic fistula. However, as the pancreas was handled atraumatically, no anastomotic leakage was observed in the past 6 years (*n* = 131).

Intracorporeal B-I reconstructions, other than DA, ART, INTACT, and Mod-DA, are characterized by complicated resection of the duodenal stump during closure of the common entry hole. Moreover, through the resection of the duodenal stump, these anastomoses are designed to reduce T-shaped stapler intersections. The main difference between each anastomotic technique is the method of closure of the common entry hole [13–18]. Both INTACT and Haung et al.’s Mod-DA [16, 17] use a single stapler to suture the closure of the common entry hole with the duodenal stump, whereas ART and Harada et al.’s Mod-DA [18] use two staplers to suture the closure of the common entry hole with the duodenal stump. Furthermore, there are two T-shaped stapler intersections in DA, compared to one in the INTACT and Mod-DA techniques of Haung et al. and Harada et al., and none for ART. Omori et al. [14], in 2013, developed INTACT, a B-I anastomotic technique that was an improved version of DA, and Yanagimoto et al. [15], in 2020, reported surgical outcomes for INTACT that included no anastomotic leakage or stenosis (n = 177). The advantages of this anastomotic method include less food residue and less residual gastritis. The authors posited that INTACT is a more physiological end-to-end anastomosis than the DA, which is a side-to-side anastomosis. Moreover, INTACT may prevent a reduction in postoperative duodenal peristalsis by resecting the ischemic or poorly perfused duodenal stump. These factors may have a favorable effect on gastroduodenal clearance of food residues in the remnant stomach [15]. Harada et al. [18] used a Mod-DA that reduced T-shaped stapler intersections, and they fired the ELS twice to remove the duodenal stump, whereby the incidence of anastomosis-related complications was 2.8% and 1.7% in the DA and Mod-DA groups, respectively [18], with less food residue and remnant gastritis, which is an advantage of Mod-DA [18]. Both researchers speculated that these anastomoses are superior to DAs for food passage because the resection of the duodenal stump prevents a reduction of duodenal peristalsis. In the present study, the ART group showed good outcomes, with −7% weight loss at the 1-year postoperative timepoint, which supports this hypothesis in food passage. Harada et al. [18] reported weight loss rates of − 8.9% and − 7% for DA and Mod-DA at the 1-year postoperative timepoint, with which our results align. The Mod-DA technique used by Harada resembles ART in that the duodenal stump was resected, and the common entry hole was sutured with two staples; however, a T-shaped stapler intersection occurred in one location. Therefore, suturing the common entry hole with two linear staplers, as in ART, may create a large anastomotic opening and, thereby, favorably affect postoperative oral intake. Zhang et al. [22] compared ART and DA and found a lower incidence of anastomotic stenosis in the ART group than in the DA group (2.25%), with less food residue in the stomach remnant in the ART group (13.3%) than in the DA group (28.2%) during endoscopy conducted at the 1-year postoperative timepoint. We speculated that ART might be advantageous for postoperative recovery and anastomotic stenosis.

Distal gastrectomy is the most commonly performed procedure for GC, and B-I and R-Y are the most commonly used reconstructive methods [26, 27]. B-I is the preferred reconstructive method after a distal gastrectomy because it is relatively simple, allows physiological food passage through the duodenum, has physiological advantages such as a reduced incidence of postoperative cholecystitis and cholelithiasis, and enables easy postoperative endoscopy and access to the papilla of Vater. However, the disadvantages of B-I include a high incidence of residual gastritis and biliary reflux [26–29]. R-Y reconstruction is superior to B-I reconstruction in the long-term results pertaining to the frequency of residual food, bile reflux, residual gastritis, reflux esophagitis, Roux stasis syndrome, and delayed gastric emptying, which may affect the patients’ postoperative quality of life [11, 26, 28, 30]. Therefore, the optimal standard reconstructive technique remains debatable, in the absence of any consensus.

To the best of our knowledge, there are four reports of ART anastomosis [13, 22, 23, 31], but none compares the surgical outcomes or postoperative nutritional status with that of R-Y. This study is the first to analyze the differences in postoperative outcomes between ART and R-Y after LDG. This study showed that, compared to the ART group, the R-Y group had a longer operative time (326 min vs. 260 min, *p* < 0.0001) and greater blood loss (98 mL vs. 32 mL, *p* < 0.0001). These results are similar to those reported previously [28]. There were no significant intergroup differences in anastomosis-related complications (ART group vs. R-Y group: 1.1% vs. 1.4%). None of the patients in the ART group experienced delayed gastric emptying, which occurred in one (1.4%) patient in the R-Y group; however, the difference was not statistically significant (*p* = 0.2). In the first postoperative year, weight loss rates of 7.0–9.3% for B-I and 9.4–10.9% for R-Y have been reported [18, 23, 28–31]. Generally, when the remnant stomach is small, R-Y is selected as the size of the remnant stomach may affect postoperative weight loss. To rule out this influence as much as possible, this study excluded patients who underwent partial gastrectomy for tumors in the U-region and had extremely small residual stomach volumes. In this study, after the first postoperative year, the ART group (–7%) had a lower rate of weight loss than the R-Y group (–7.9%) although the difference was not statistically significant (*p* = 0.905). These results indicated that the ART anastomosis was relatively effective in terms of postoperative oral intake. In this study, the ART and R-Y groups had similar nutritional statuses at the 1-year postoperative timepoint. In a large-scale multicenter cohort study by Kinoshita et al. [28] that compared weight loss and nutritional parameters (e.g., albumin, total protein, and lymphocyte count) at 6 months, 1 year, 3 years, and 5 years, the only statistically significant difference between the B-I and R-Y groups was the serum total protein level at the 1-year timepoint (*p* = 0.0001). From a long-term nutritional perspective, the R-Y reconstruction is nearly equivalent to the B-I reconstruction. In a multi-institutional RCT by Kimura et al. [29], there was no difference between B-I and R-Y reconstruction in terms of weight change, nutritional status (albumin–lymphocyte count, PNI), or quality of life, not only at 1 year but also at 5 years postoperatively. R-Y reconstruction after gastric resection was superior to B-I reconstruction in preventing remnant gastritis and lower esophagitis [26]. Compared to the B-I group, the R-Y group had a lower long-term incidence of bile reflux into the remnant stomach and reflux esophagitis. Furthermore, the nutritional status and late complications did not differ between the R-Y and B-I groups [12]. In general, post-gastrectomy weight loss is significant in the early postoperative period and stabilizes after 6 to 12 months [27, 28], and this was confirmed in this study, wherein the lowest rate of weight loss occurred at 6 months postoperatively in both the ART and R-Y groups.

In this study, a detailed examination of postoperative endoscopic findings was not performed; however, the low rate of postoperative weight loss as compared with other intracorporeal B-I reconstructions suggests that post-ART food passage may have been favorable. Furthermore, previous reports have included results from patients who were undergoing postoperative chemotherapy, and the side effects of anticancer drugs may also have affected the weight and nutritional status due to anorexia. Thus, to assess the exact postoperative weight loss rate and changes in nutritional status, we excluded patients who received postoperative adjuvant chemotherapy. Weight loss after gastrectomy has been associated with compliance with postoperative chemotherapy [32], and reconstruction methods with a lower rate of weight loss, such as the approach described in this study, are thought to contribute to longer survival rates.

This study had some limitations. First, this was a single-center retrospective study with a small sample size, and a potential selection bias could not be excluded. Second, ten surgeons performed the surgeries in this study. However, in all cases, surgeons with board certification in gastrointestinal and endoscopic surgery operated as surgeons or first assistants. Third, the study period was only 10 years. During the study period, the surgeons’ laparoscopic techniques, surgical instruments, and methods of developing the surgical field gradually changed. In particular, in the last 6 years, methods that do not compress the pancreas have been introduced to prevent pancreatic fistulas owing to the effects of pancreatic direct compression in suprapancreatic lymph node dissection. However, the techniques and devices used for both anastomotic methods have not changed. Future multicenter, prospective, randomized controlled studies should be conducted to determine the safety and efficacy of ART anastomosis after LDG for GC.

In conclusion, ART can be performed safely with a low incidence of anastomosis-related complications. Furthermore, ART facilitates reconstruction of a large quadrilateral end-to-end anastomosis, which has superior outcomes in terms of postoperative food intake. To verify the benefits of ART after LDG, it is necessary to evaluate the short- and long-term outcomes in a large number of patients.

## Supplementary Information

Below is the link to the electronic supplementary material.

Supplementary file1 (MP4 185131 KB)
